# Applying Broadband Dielectric Spectroscopy (BDS) for the Biophysical Characterization of Mammalian Tissues under a Variety of Cellular Stresses

**DOI:** 10.3390/ijms18040838

**Published:** 2017-04-15

**Authors:** Maria P. Souli, Panagiotis Klonos, Adamantia F. Fragopoulou, Ifigeneia V. Mavragani, Ioannis S. Pateras, Nikolaos Kostomitsopoulos, Lukas H. Margaritis, Pavlos Zoumpoulis, Loukas Kaklamanis, Dimitris Kletsas, Vassilis G. Gorgoulis, Apostolos Kyritsis, Polycarpos Pissis, Alexandros G. Georgakilas

**Affiliations:** 1 Physics Department, School of Applied Mathematical and Physical Sciences, National Technical University of Athens, Zografou Campus, 15780 Athens, Greece; mariasouli@mail.ntua.gr (M.P.S.); pklonos@central.ntua.gr (P.K.); ifimav@mail.ntua.gr (I.V.M.); akyrits@mail.ntua.gr (A.K.); ppissis@central.ntua.gr (P.P.); 2 Department of Cell Biology and Biophysics, Faculty of Biology, University of Athens, 15701 Athens, Greece; madofrag@biol.uoa.gr (A.F.F.); lmargar@biol.uoa.gr (L.H.M.); 3 Molecular Carcinogenesis Group, Department of Histology and Embryology, School of Medicine, University of Athens, 11527 Athens, Greece; ipateras@med.uoa.gr (I.S.P.); vgorg@med.uoa.gr (V.G.G.); 4 Laboratory Animal Facilities, Center of Clinical, Experimental Surgery and Translational Research, Biomedical Research Foundation, Academy of Athens, 4 Soranou Efesiou Street, 11527 Athens, Greece; nkostom@bioacademy.gr; 5 Diagnostic Echotomography Medical S.A., 317C Kifissias Avenue, 145 61 Kifissia, Greece; p.zoumpoulis@echomed.gr; 6 Department of Pathology, Onassis Cardiac Surgery Center, 356 Sygrou Avenue, 17674 Kallithea, Athens, Greece; loukasgka@yahoo.gr; 7 Laboratory of Cell Proliferation and Ageing, Institute of Biosciences and Applications, National Centre for Scientific Research “Demokritos”, 60037 Athens, Greece; dkletsas@bio.demokritos.gr

**Keywords:** broadband dielectric spectroscopy, radiation, tissues, cancer, cellular stress, genomic instability

## Abstract

The dielectric properties of biological tissues can contribute non-invasively to a better characterization and understanding of the structural properties and physiology of living organisms. The question we asked, is whether these induced changes are effected by an endogenous or exogenous cellular stress, and can they be detected non-invasively in the form of a dielectric response, e.g., an AC conductivity switch in the broadband frequency spectrum. This study constitutes the first methodological approach for the detection of environmental stress-induced damage in mammalian tissues by the means of broadband dielectric spectroscopy (BDS) at the frequencies of 1–10^6^ Hz. Firstly, we used non-ionizing (NIR) and ionizing radiation (IR) as a typical environmental stress. Specifically, rats were exposed to either digital enhanced cordless telecommunication (DECT) radio frequency electromagnetic radiation or to γ-radiation, respectively. The other type of stress, characterized usually by high genomic instability, was the pathophysiological state of human cancer (lung and prostate). Analyzing the results of isothermal dielectric measurements provided information on the tissues’ water fraction. In most cases, our methodology proved sufficient in detecting structural changes, especially in the case of IR and malignancy. Useful specific dielectric response patterns are detected and correlated with each type of stress. Our results point towards the development of a dielectric-based methodology for better understanding and, in a relatively invasive way, the biological and structural changes effected by radiation and developing lung or prostate cancer often associated with genomic instability.

## 1. Introduction

There is a great need for the discovery of non-invasive methodologies to determine structural changes in vivo, or at least ex vivo in tissues after exposure to a type of environmental stress. In this case, we refer to environmental stresses, such as ionizing (IR) and non-ionizing radiation (NIR), chemicals, etc. Radio frequency electromagnetic radiation (RF-EMR) emitted by various wireless devices used in everyday life, like mobile phones, DECT (Digital Enhanced Cordless Telecommunication) phones, and Wi-Fi routers, have been considered as a stress factor for living organisms. In a population-based study, health complaints, like tiredness, stress, headache, anxiety, concentration difficulties, and sleep disturbances, were reported after exposure to wireless phones [1]. In a pioneering proteomics study, Fragopoulou et al. showed that the expression levels of stress-related proteins (heat shock proteins, HSP) were increased, among others, in the mouse brain after exposure to DECT or mobile phone radiation [2]. The same group has also reported reactive oxygen species (ROS) levels increase in the bodies and ovaries of *Drosophila melanogaster* after exposure to DECT-based radiation [3]. Exposure to RF-EMR has also elicited an HSP response in rat brains [4,5]. Thus, it is postulated that the pulsed idle state of the DECT-based radiation (pulse duration of 0.08 ms at a rate of 100 Hz) may act as an environmental stimulus capable of producing stress responses, albeit at a very low specific absorption rate (SAR) for DECT radiation. On the other hand, IR (like X- or γ-rays) is capable of ionizing molecules and atoms by freeing electrons and, thus, producing a cascade of damaging events for biological material, like cells and their valuable content (DNA, lipids, and proteins). The induction of complex (highly-localized) DNA damage is considered the signature of any IR and the basis of its detrimental effects in every living tissue and organism, and the characterization of IR as a genuine and severely harmful environmental stress [6]. In this category of stressors, we can also include cancer and malignant diseases, in general, since they are usually associated with an environmental impact and, although not really exogenous to the organism, they formulate a type of stress in the cell or tissue. Recently, and based on accumulated evidence, a unifying model was suggested underlying the similar phenotypes and responses observed between advancing malignancy and IR [7]. Additionally, at the organism level, these initial effects may be amplified by systemic actions inducing DNA damage in distant sites of the human body as a result of inflammatory and immune response [8].

There is always a necessity of accurate and non-invasive techniques when it comes to tissue damage. One of the less-invasive biophysical approaches considered is dielectric spectroscopy, and it has been utilized by different laboratories in order to measure characteristics either of DNA in solution, proteins, or even tissues [9,10,11,12,13,14,15,16,17]. Our laboratory has specifically applied broadband dielectric spectroscopy (BDS) to measure DNA and cellular damage after exposure to ionizing radiation, apoptotic cells and, in general, as a sensitive damage biosensing methodology [18,19]. BDS in this case, has been compared with a series of independent techniques, such as ultraviolet thermal transition spectroscopy, pulsed field gel electrophoresis, multiple microgel “comet assay”, and scanning electron microscopy. Using these techniques, the induction of DNA degradation and cell membrane disruption in human peripheral blood mononuclear cells was investigated underlying the emerging potential of BDS for following structural and physicochemical changes in the DNA (breaks, base damage, etc.) and cellular responses to DNA damage, like apoptosis (programmed cell death), etc. [20]. Dielectric properties of tissues have been investigated in the past and applied, for example, in the discrimination and possible diagnosis of normal to malignant tissues and other histopathophysiological differences, and even underlying molecular mechanisms [21,22]. The idea lies on the fact that structural tissue differences, such as that in water content, membrane integrity, membrane lipid percentages, and cellular state, such as resting or proliferative cells, can result in significantly different electrical properties and, consequently, dielectric response, etc. [10,22,23]. In this work we used BDS for the initial characterization of a variety of rat tissues’ responses to ionizing and non-ionizing radiation and to compare malignant to non-malignant human lung and prostate tissues. Our results underline the possible applicability of BDS as a non-invasive technique for the detection of physicochemical tissue changes due to radiation or malignant transformation [24,25].

## 2. Results

### 2.1. Broadband Dielectric Spectroscopy (BDS) Measurements

**Figure 1:**
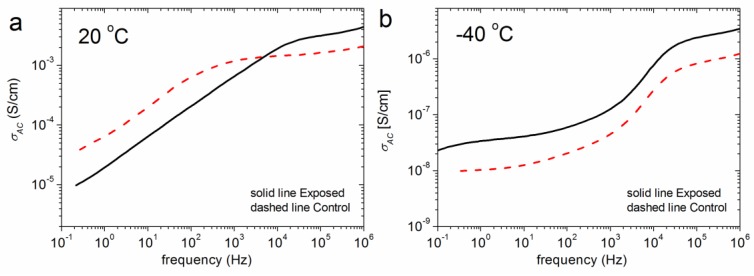
Comparative broadband dielectric spectroscopy (BDS) spectra of alternating current conductivity, σ_AC_, (**a**) at 20 °C and (**b**) at −40 °C for rat cerebellum. The solid line represents a rat sample that is exposed to DECT (Digital Enhanced Cordless Telecommunication) prenatally, plus one month postnatally, while the dashed line (---) represents the control.

Our preliminary study includes various types of tissue, in sets of control versus malignant, excised from both humans (lung and prostate) and rats (cerebellum and skin). Each sample was subjected into BDS measurements, according to the below-mentioned protocol. In all cerebella cases studied, as the frequency increases, AC conductivity, σ_AC_, presents a step-like increment, revealing a plateau at frequencies around 10^5^ Hz at 20 °C and 1 Hz at −40 °C (Figure 1). The values of AC conductivity at this plateau area is considered as DC conductivity σ_DC_, and are presented in Table 1.

**Figure 2:**
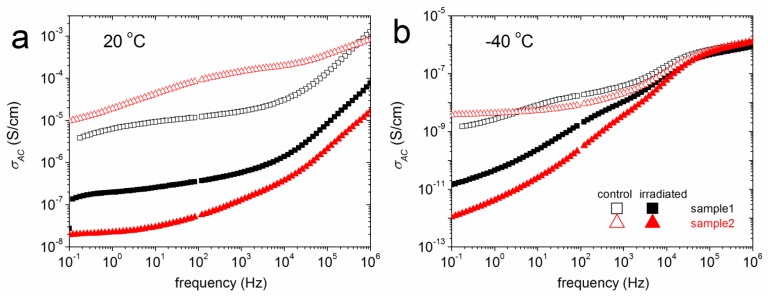
Comparative BDS spectra of AC conductivity, σAC, (**a**) at 20 °C and (**b**) at −40 °C of rat skin. The solid symbols (▪) represent rat skin samples that are exposed to γ-radiation, while the open symbols (□) represent the control.

Considering the vast effect of γ-radiation on tissues, rat skin obtained from animals exposed to a dose of 5 Gy and total body γ-irradiation (Figure 2), was studied and found in both cases to be less conductive than normal tissue at ambient temperature (Table 1). The recorded conductivity spectra shows a possible difference in structure of irradiated skin at low frequencies when the tissues are in a frozen state (Figure 2b).

Results with cancerous human lung tissues are also presented in Table 1. In these preliminary results, healthy tissues show greater values of conductivity at 10^5^ Hz. On the other hand, human prostate cancer tissue with a Gleason score of 6 appears to be more conductive than the control (healthy) ones during the whole cooling procedure, while more aggressive forms of cancer (Gleason score 8) seem to be less conductive than the control ones.

### 2.2. Tissue Water Fraction

Tissue hydration on a wet basis, *h*, and during BDS measurements, *h*_BDS_, calculated through Equations (1) and (2) are included in Table 2. The values of *h* are in satisfactory agreement with literature values [26] and no important diversity was recorded. The values of *h*_BDS_ show that there is 11–25% water loss during the cooling and heating procedure.

**Figure 3:**
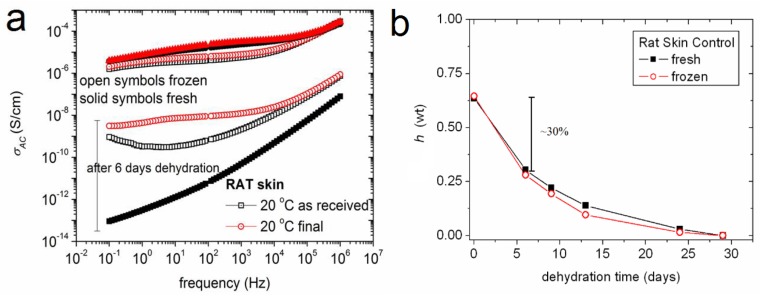
Comparative BDS spectra of AC conductivity, σ_AC_, for fresh and frozen tissues before and after six days of dehydration (**a**) and the decrease of water fraction, *h*, during dehydration by exposure to a low water vapor atmosphere (**b**). Within six days almost 30% of the total water fraction of the skin tissue is lost. This results in recording a dramatic drop in AC conductivity.

In order to measure the effect of the water fraction on the dielectric conductivity, the geometrical stability of rat skin tissues and the capacitors formed by them were exploited so as to perform repetitive measurements. Rat skin tissues, one freshly excised and a second kept under refrigerated (4 °C storage, were measured in a hydrated state and after six days of dehydration. In Figure 3a, AC conductivity spectra of both tissue states (fresh/frozen) before and after dehydration are included. Furthermore, conductivity at 20 °C is presented twice for each sample, i.e., in the beginning of the experiment (as received) and in the end of the thermal circle (final). No significant difference was found between fresh and frozen tissues during the first measurement. A profound decrease of conductivity is recorded for tissues with lower hydration levels. In Figure 3a are presented the results for the conductivity after a six-day dehydration time, which is almost equal to 30% water loss from the tissues (Figure 3b). No further analysis could be performed on the tissue structure at ambient temperature. The next step was to focus on below-zero temperatures and study the relaxation times as recorded by isothermal measurements during heating from −150 to 20 °C.

### 2.3. BDS Analysis

**Figure 4:**
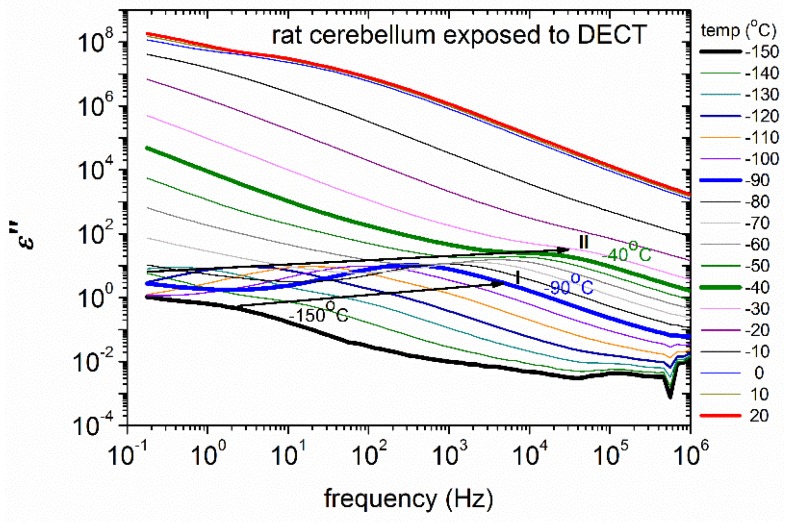
The recorded dielectric spectra of ε" of rat cerebellum exposed to DECT (raw data). Black arrows are used to emphasize the existence of Havriliak-Negami terms. Relaxations I and II are easily identified. Additional relaxation processes are identified only by analysis. The results of the analysis are presented in the Arrhenius plot (Figure 5).

Isothermal dielectric measurements in a broad temperature range were performed in different tissues of control rats with the aim to test the strength of the BDS method applied on biological matter. Measurements at subzero temperatures (Figure 4) revealed a wide variety of relaxation processes. Further results by means of analysis (HN equation, Equation (3)) are explicated in the Materials and Methods section below.

## 3. Discussion

### 3.1. BDS Measurements

The dielectric spectra of the tissues studied herein follows the typical frequency dependence of the complex permittivity and conductivity of a heterogeneous material, as described in the relevant literature [10]. The step-like increment in AC conductivity, σ_AC_, of all of the cerebella cases studied, and the plateau that is formed at frequencies around 10^5^ Hz at 20 °C and 1 Hz at −40 °C (Figure 1), is a typical conductivity trend for this type of tissues [27]. This behavior is ascribed to different phenomena that take place due to the consistency of 80% water content (Table 2), the structure of the tissue itself, and the way this combination behaves in a frozen state. The conductivity values of all tissues studied at these particular frequencies and temperatures are presented in Table 1.

Concerning the type of stress and tissue, the information regarding rat cerebellum exposed to different doses of DECT does not preclude, at this stage, univocal conclusions. However, this could be due to the mild, and in many cases unknown, effects imposed by DECT radiation (non-ionizing) on tissues, as compared to those caused by γ-radiation (extensive tissue and cellular damage [28,29]).

Considering the effect of γ-radiation on rat skin obtained from animals exposed to a dose of 5 Gy (Figure 2), the irradiated tissues were found less conductive than normal ones at ambient temperature (Table 1). Conductivity values of irradiated and control skin are in agreement with earlier studies [30]. The recorded conductivity spectra show a possible difference in structure of irradiated skin at low frequencies when the tissues are in a frozen state (Figure 2b).

According to results with cancerous human lung tissues a strong connection to histopathological characteristics, such as to those of tissue necrosis, is obtained. These first results evince the possible satisfactory resolution of the BDS technique towards the detection of malignancy. This change may have an effect on ion channels during signal transduction or other conductivity-dependent processes. In addition, as discussed by previous studies, this may be related to the different water and fat content for cancerous tissues [22,23] and for irradiation to the cell (nuclear and membrane damage), and in general, changes in tissue structure and characteristics. In the case of IRs, we cannot exclude some systemic effects in the animals since measurements were performed 48 h after the initial exposure [7].

### 3.2. Tissue Water Fraction

Tissue hydration levels (Table 2) on a wet basis, h, are in satisfactory agreement with literature values [26], since the aim of the procedure is not the accurate definition of the water content of the tissues, but to confirm the safety of the tissues during transfer and storage. No important diversity was recorded. This fact shows that conductivity values at 20 °C measured as received (before heating/cooling) can be considered rather accurate at the present stage of this work. On the other hand, *h*_BDS_ values show that there is 11–25% water loss during the BDS measurement, something that deprives the repeatability of thermal circle experiments. Furthermore, the h and *h*_BDS_ values for control rat skin that was measured right after resection were similar to those that were stored at below-zero temperatures. This allows further experimentation with frozen tissues, a fact that simplifies the protocol and the timeline of such experimental procedures.

Focusing on the rat skin and comparing the dielectric spectra of fresh and frozen tissues during the first measurement (Figure 3a, marked “as received”) no significant difference was found between them. This suggests that the BDS can also be applied to frozen tissues, like biopsies. Dehydration in all cases results in the decrease of conductivity. In the case of a six-day dehydration time, almost 30% of water is lost from tissues (Figure 3b), something that results in the aforementioned conductivity decrease and/or structural changes recorded as the dielectric behavior of an insulator, like fresh tissue after six days of dehydration (Figure 3a).

### 3.3. BDS Analysis

**Figure 5:**
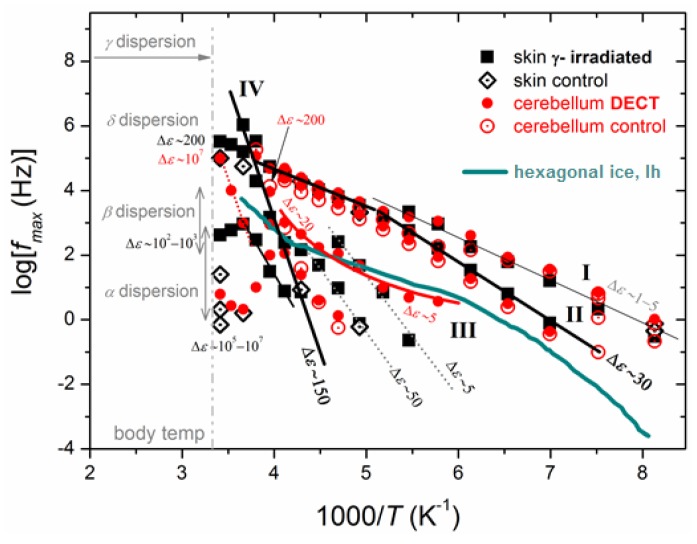
Arrhenius plots of the recorded relaxations for irradiated and control rat tissues. Black symbols refer to skin which is either exposed to γ radiation (■) or intact control ones (♢). Red symbols refer to the cerebellum, either exposed to DECT (●) or control ones (○). The black drawn lines are used as guides to the eyes. Included in the plot is the time scale of bulk hexagonal ice [33], *Ih* (solid blue line). Results from the respective literature for α, β and γ dispersions (details in text) in biological tissues [10,11,37] have been comparatively added at body temperature (~35 °C). The values for the dielectric strength, Δε (Equation (3)), for the relaxations recorded are also included in the plot.

Isothermal dielectric measurements at subzero temperatures in different tissues of control rats (Figure 4) provide information on the water fraction that crystallizes and on the water molecules that exhibit supercooling and do not self-organize in a crystalline structure (uncrystallized water). It is known that interfacial or biological water molecules avoid crystallization and adopt structures and dynamics that depend on their specific interactions and their spatial confinement [31]. Furthermore, it is realized that supercooled and glassy water are intimately related, and that fundamental understanding of thermodynamics and transport properties of biological water must encompass both its liquid and vitreous states [32]. BDS measurements can shed light on specific features of the hydrogen bonding network of water molecules within the tissues. Data analysis revealed the activation of various polarization processes in rat tissues. In Figure 5 we present the so-called Arrhenius plot for the temperature dependence of the time scale of the detected relaxation processes. The data obtained from the control tissues are also included. The spectra shown in Figure 4 and the plots in Figure 5 reveal that many relaxation processes are activated in the temperature range studied. Four relaxation processes are indicated on the plots and their underlying molecular mechanisms are briefly discussed. Relaxations I and II appear at low temperatures and may be related with the crystalline ice phase. For comparison, the Arrhenius plot of the bulk hexagonal ice, *Ih*, is also shown in Figure 5. We observe that relaxation II has similar activation energy with ice *Ih* at low temperatures, while relaxation I exhibits a slightly lower activation energy. In addition, data analysis reveals that the dielectric strength of relaxation II is rather high, Δε~30, comparable with the dielectric strength of the ice phase in bulk [33,34], while relaxation I has smaller dielectric strength (Δε~1–3). Therefore, we attribute relaxation II to the ice *Ih* phase whereas (the faster) relaxation I may be related with polarization in the disordered liquid-like surface layers of the ice crystallites [35]. The fact that the time scale of relaxation II shows different temperature dependence than those of bulk hexagonal ice has also been observed in other hydrated systems and reflects, actually, the great sensitivity of the dielectric properties of ice crystallites on their defects and on the impurities that are incorporated in their lattice [34,36]. It is interesting that all of the tissues exhibit relaxations I and II with similar characteristics. On the contrary, relaxation III seems to appear only in stressed tissues. The location of relaxation III in the plots of Figure 5 implies that it is related with the polarization of bulk ice *Ih* that is formed within stressed tissues at temperatures above *T* = −100 °C. Finally, at temperatures higher than about −40 °C relaxation IV appears in all of the obtained spectra. Relaxation IV has high dielectric strength (Δε~150–200) and high activation energy. These features suggest that this process may be related with the interfacial polarization process due to enhanced protonic conductivity in this temperature range within clusters of uncrystallized water molecules (since the trace of this process is unaffected by ice melting). Worth noticing is that the extrapolation of the trace of relaxation IV to the body temperature region points to γ-dispersion that is usually observed in high-frequency dielectric measurements on non-frozen tissues. Additional relaxation processes that appear in the left part of Figure 5, i.e., at high temperatures, may be related with additional interfacial polarization processes due to high ionic conductivity and the remarkable heterogeneity of the tissues.

The results were compared with previous data for higher frequencies (extended black lines in Figure 5) and were found consistent with previous work [13]. Four main dispersions arise in the analysis, α, β, γ, and δ dispersions. α-Dispersion is attributed to ionic diffusion processes in charged membranes [38], endoplasmic reticulum [39], cellular membrane’s potential, and counterions’ displacement around them [40]. β-Dispersion is correlated with the cellular plasma membranes’ interfacial polarization and electrolytes of the intra- and extra-cellular space [13]. γ-Dispersion is associated with the water component of biological species and small molecules [41]. The special characteristics of the biological matter spectra, such as biopolymers, cellular organelles [40], proteins, and protein-bound water [42], is δ-dispersion. First characterized by Pethig (1984) [9], δ-dispersion is identified to be a group of sub-dispersions between the β- and γ-dispersion, so faint that it can only observed during the analysis process. δ-Dispersions are due to the large molecules’ dipolar moments (proteins).

## 4. Materials and Methods

### 4.1. Animal Exposure

For the non-ionizing radiation exposure, animal experimentation was performed in the Biomedical Research Foundation of the Academy of Athens (BRFAA) and protocols were evaluated and approved on 24-11-2011 by the Veterinary Service of the Prefecture of Athens (permit number 4215/24-11-2011). All experiments were performed in accordance with the approved guidelines and the Greek Presidential Decree 56/2013, which harmonizes national legislation with the European Community Directive 63/2010 on the Protection of Animals Used for Scientific Purposes. Wistar rats were whole-body exposed during prenatal only, or pre- and post-natal life (up to one month postnatally) for 12 h/day (intermittently; half an hour ON and half an hour OFF), to DECT-based radiofrequency electromagnetic radiation (RF-EMR) at 3.7 V/m average electric field intensity, as previously described [43]. No mortality was detected. Rats were euthanized by CO_2_ inhalation using a euthanasia chamber. The flow rate of CO_2_ was gradually increased up to 6 L/min. Brain tissues (cerebellum) were immediately isolated and snap frozen in dry ice. Then, the tissues were stored at −80 °C until further processing. Due to the vulnerability of this type of tissue (high water content) and the fact that the morphology of the cerebellum is well-defined by an external membrane, no intervention on the volume was made, in order to be able to keep this structure as intact as possible. The mass of cerebellums was 1–2 g and the diameter was less than 1 cm (considered as sphere-like-shaped).

For ionizing radiation exposures, two-month old Wistar albino rats were exposed to 5 Gy of γ-radiation in a ^60^Co γ source (Gamma Chamber 4000 A, Isotope Group, Bhadha Atomic Research Company, Trombay, Bombay, India) at a rate of 3.2 Gy/min for total ~1.5 min (94 s) and were housed in the Animal House of The Institute of Biosciences and Applications of NCSR “Demokritos” and the protocol approved by Institutional animal ethical committee (animals were treated under license 6455). This dose is much lower than the expected LD_50/30_ dose (8–9 Gy) of low-LET radiation like X- and γ-rays, i.e., the mean lethal dose that causes 50% death in animals exposed to the radiation after 30 days [44,45]. Two days after irradiation, and with no mortality or any other sings of organ malfunction detected, the animals were sacrificed and skin samples were taken and stored immediately at −20 °C. Rat skin was carefully excised from the animals, minimizing fat tissue inclusion. Since the surfaces of tissues were quite large, they were cut properly (with a sterile surgical blade) to fit in a 25 mm-diameter brass plate electrode. 

### 4.2. Human Malignant Tissues

Human lung tissues were obtained from the Laboratory of Histology and Embryology, Athens Medical School, University of Athens, after lung resection surgical procedures on patients diagnosed with cancer. These sets of samples consist of a part of the carcinoma and a part remote from the maleficence. All samples were collected according to local ethical committee approvals. None of the patients had undergone any cancer therapy before surgical resection of the lesions. The tissues were stored at −80 °C and their size (after defrosting and properly cut with sterile surgical blade) was approximately 1 cm in diameter and 2–4 mm in thickness. Cryosections for hematoxylin and eosin staining were taken before BDS measurements, in order to obtain a view of the condition of each tissue.

Human prostate tissues were obtained from eighteen-core needle biopsies performed on men who had been diagnosed with prostate cancer. Biopsies were carried out at Diagnostic Echotomography SA and the tissues were stored at −20 °C. The volume was a cylindrical sample of ~1 mm in diameter. Adjacent biopsies were sent for histological examination and the condition of each patient was evaluated.

Specifically, BDS was applied on two (2) sets (healthy versus malignant) of human lung and three (3) sets of human prostate tissues, and two (2) sets (control vs. irradiated) of rat skin are included in this study. Furthermore, a set of two rat skin tissues, consisting of an immediately-excised tissue and one that was stored at −80 °C, was measured in order to investigate the freezing of free and semi–bound water molecules (ice) in the systems [12]. Four (4) control and five (5) samples exposed to DECT rat cerebella are included in this study, which are grouped and compared according to each irradiation conditions. One set of each type of tissue was also subject to the specific drying procedure described below in order to define the total water fraction of tissues, h, and the water fraction after the experiment, *h*_BDS_, with the aim to ensure the sufficiency of the protocol on the structure vulnerability through a comparison with the literature. In addition to all of the above, and as there is a clear analogy between biological matter and other soft materials, a liver lobe, a cerebellum, a half brain, and a hippocampus of a control rat were also studied so as to observe the dynamics of different tissues from a material physics approach.

### 4.3. Broadband Dielectric Spectroscopy (BDS)

For BDS measurements each sample was inserted between two finely-polished brass disk-like plates of a capacitor for dielectric measurements. A plate of 4 or 25 cm diameter was used as a base for the tissues and a second smaller brass plate was placed on top of the sample to form a parallel plate capacitor. The diameter of the top plate was varied and is the one that defines the size of the capacitor. The two plates were chosen to be lightweight to avoid extra stress and strain of the samples. A slight pressure was imposed on the top electrode by hand, only to secure proper tissue-metal contact. As far as there was no fixation, there was no need of any special preparation, except in cases of blood on a tissue, when a quick immersion in distilled water took place and the tissue was left to drain from extra moisture on a piece of paper on a Petri dish. Rat skin, as a well-defined film, was easily placed between finely-polished brass electrodes, forming capacitors of 25 mm diameter and ~2 mm thickness. Rat cerebellum and human lung tissues need more delicate handling, as sometimes a wash was necessary to remove blood. Those tissues filled capacitors of 8 mm diameter (the size of the top electrode) and ~2–3 mm thickness with gentle pressure to secure good contact and proper parallel geometry. Prostate tissues were the most difficult to handle, due to their complicated geometry and, therefore, the smallest possible electrode was used (with a diameter of 8 mm) and the capacitor thickness was ~0.2–0.7 mm.

Then the capacitor is placed in a Novocontrol sample cell, an alternating voltage was applied between electrodes, and the conductivity, σ_AC_, and the complex dielectric permittivity, ε*°=°*ε*′°−°*iε*″, were recorded isothermally (in nitrogen atmosphere) as a function of frequency in the range from 10^−1^ to 10^6^ Hz at temperatures from 20 to −150 °C on cooling and, subsequently, from –150 to 25 °C on heating in steps of 5 and 10 °C using a Novocontrol Alpha Analyzer. A Novocontrol Quatro Cryosystem controlled the temperature to better than 0.5 °C.

### 4.4. Determination of Water Fraction, h

A specific drying procedure was employed in order to answer questions about the water content of the tissues. Samples were initially equilibrated at ambient conditions. In order to evaluate the ambient hydration level (water content) samples were dried by exposing them to a low water vapor atmosphere in sealed jars [46]. The relative humidity rh ~0.02 was achieved with phosphor pentoxide, P_2_O_5_, in the form of saturated aqueous solution. The attainment of equilibrium was determined via recording of the sample mass (*m*_sample_). A Mettler Toledo balance with 10^−5^ g sensitivity was employed for these measurements. The mass of the sample before the BDS measurement was consider as the mass in a hydrated state (*m*_hydrated,sample_), while the mass of the sample after equilibration over P_2_O_5_ was considered as the mass in dry state (*m*_dry,sample_). Once the equilibrium was attained, the hydration, *h*, was calculated on a wet basis via Equation (1):

(1) $$h=\frac{m_{water}}{m_{hydrated,sample}}=\frac{m_{hydrated,sample}-m_{dry,sample}}{m_{hydrated,sample}}$$

In order to define the water rate of tissues after their thermal “annealing” by freezing at −150 °C and heating back to 20 °C, hydration during BDS measurement, *h*_BDS_, was calculated on a wet basis by Equation (2):

(2) $$h_{BDS}=\frac{m_{water}}{m_{hydrated,sample}}=\frac{[(m_{hydrated,sample}-m_{afterBDS})/2]-m_{dry,sample}}{m_{hydrated,sample}}$$

where *m*_afterBDS_ is considered as the mass of the sample as measured right after the BDS experiment.

### 4.5. Dielectric Data Analysis

**Figure 6:**
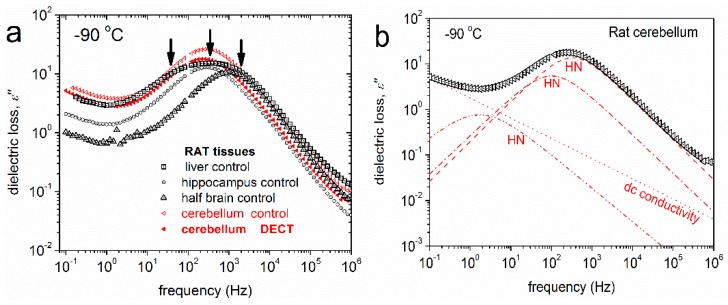
(**a**) The imaginary part of dielectric permittivity, *ε*", against frequency for various rat tissues at −90 °C. The arrows point at the frequency of maximum dielectric loss for the recorded relaxation mechanisms; and (**b**) An example of the analysis procedure at −90 °C, in terms of individual HN (Equation (3)) components, for each of the segmental relaxations.

BDS results were analyzed by fitting model functions [47] to the experimental data employing appropriate software [48] in order to evaluate the time scale (temperature dependence of the frequency maxima of dielectric loss, *ε’*) (Figure 6a), the dielectric strength, and the shape parameters of the recorded relaxations [49]. To that aim we employed the asymmetric Havriliak–Negami (HN) equation:

(3) $$\varepsilon^{*}\left(f\right)=\varepsilon_{\infty }\;+\frac{\Delta \varepsilon }{{\left(1+{\left(if/f_{0}\right)}^{\alpha_{HN}}\right)}^{\beta_{HN}}}$$

HN terms of Equation (3) for each of the relaxations were critically fitted to the experimental data at each temperature (Figure 6b) and the fitting parameters were determined. The number of terms varied for different types of tissue and temperatures, depending on the number of relaxations present and the extent of their overlapping. In Equation (3), ε_∞_ describes the value of the real part of dielectric permittivity, ε′, for *f* >> *f*_0_, Δε is the dielectric strength, *f*_0_ is a characteristic frequency related to the frequency of maximum dielectric loss (ε"), and α_HN_ and β_HN_ are the shape parameters of the relaxation. We recall that the deviation of β_HN_ from 1 describes the asymmetry of the relaxation, whereas the deviation of α_HN_ from 1 describes the broadening of the relaxation. Thus, the symmetric Debye relaxation with a single relaxation time is characterized by α_ΗΝ_ = 1, β_ΗΝ_ = 1.

Employing such an analysis on the dielectric spectra recorded by measuring rat tissues (stressed and control) at broad temperature ranges we were able to construct Arrhenius plots (Figure 5). Such plots show the temperature dependence of the time scale of each distinct polarization mechanism activated in the tissues and, therefore, provide information on the activation energies of the underlying molecular mechanisms.

## 5. Conclusions

The contrast and, at least in most cases, systematic difference in electrical conductivity between different tissues, depending on their architecture, morphology, and pathophysiology state, show promise for distinguishing pathogenicity and damage compared to healthy/control counterparts. The trends found suggest the possibility of different dielectric parameters for irradiated and cancerous tissues. The variability observed in some cases in our findings suggest, at the same time, that further studies should be applied with the aim of establishing a standard protocol, to exploit the sensitivity of the method and determine specific quantified thresholds for each pathogenic state usually synonymous with high genomic instability. Earlier dielectric studies on human breast and colon normal and malignant tissues, at much higher frequencies, though, suggest an increase of conductivity in the malignant state [50,51]. Although we also detected a similar trend with our prostate samples (liquid biopsies), this is not verified for our lung samples, probably due to different tissue structures and/or tissue acquisition conditions. Interestingly, dielectric properties (conductivity and relative permittivity) of excised rat lung tissue are significantly modified by lung air and tend to decrease with increasing lung air content [52]. In addition, the higher content of fat and adipose tissue in normal tissues reducing the conductivity is a very important parameter which may influence the results [17]. Therefore, we believe that, in the future, analytical studies should be performed in tissues of specific stages and types of cancer (ideally with identical water and fat content) in order to determine possible differences in the molecular dynamics of “stressed” tissues in comparison with their healthy counterparts. In addition, we believe that one of the vulnerabilities of our approach is tissue heterogeneity, which is always a potential obstacle to overcome.

Finally, it becomes clear by this work that there is great potential for the use of spectroscopic methods, such as broadband dielectric spectroscopy for medical/clinical applications. As these techniques can easily turn into portable devices, ready for use out of the laboratory environment in a non-invasive way, they could be applied as pre-histological diagnostic methods and, therefore, turn into powerful medical tools in the service of public health. Along the same lines, the BDS method can supply information about the impact of environmental stress (radiation and diseases) on living organisms, using simple physical magnitudes (conductivity) as markers. As there is a strong structure-dependency in a functional base, such information can be used in the direction of mapping the biological paths of various illnesses. The logic of combining different tissues and laboratories was to aid in a better understanding of the different types of stressors in different biological systems. The significance of our results can be regarded as a proof of concept for the application of a traditional biophysical methodology towards current developing theories and the need of non-invasive diagnostics at the organism/tissue level of the effects of an environmental stress.

## Figures and Tables

**Table 1:** The values (raw data) of conductivity, σ_DC_, at 10^5^ Hz at 20 °C and at 10^5^ Hz and 1 Hz at −40 °C, where the plateau is formed in most cases.

Conductivity, σ_DC_ (S/cm)			20 °C at 10^5^ Hz	−40 °C at 10^5^ Hz	−40 °C at 1 Hz
Tissue	Type of Stress	Dose (age)			
**Rat Cerebellum**	**DECT Exposure**	Prenatally (three months old)	1.40 × 10^−3^	5.30 × 10^−7^	5.00 × 10^−9^
			1.30 × 10^−3^	7.30 × 10^−7^	5.00 × 10^−9^
		Prenatally + one month after birth (one month old)	3.10 × 10^−3^	2.40 × 10^−6^	3.40 × 10^−8^
			1.20 × 10^−3^	7.90 × 10^−7^	4.40 × 10^−9^
		Prenatally + one month after birth (two months old)	1.00 × 10^−3^	7.20 × 10^−7^	1.00 × 10^−8^
	**Control**	(three months old)	1.50 × 10^−3^	4.70 × 10^−7^	3.80 × 10^−13^
		(one and two months old)	1.10 × 10^−3^	6.20 × 10^−7^	6.60 × 10^−9^
			1.60 × 10^−3^	8.20 × 10^−7^	1.00 × 10^−8^
			1.40 × 10^−3^	6.70 × 10^−7^	5.60 × 10^−9^
**Rat Skin**	**γ-Irradiation**	5 Gy	8.30 × 10^−6^	4.50 × 10^−7^	4.40 × 10^−11^
			2.10 × 10^−6^	5.30 × 10^−7^	4.10 × 10^−12^
	**Control**		3.60 × 10^−4^	6.20 × 10^−7^	4.20 × 10^−9^
			1.40 × 10^−4^	6.70 × 10^−7^	2.60 × 10^−9^
**Human Lung**	**Adenocarcinoma of low differentiation**	~25% necrotic cells (I)	1.90 × 10^−4^	1.40 × 10^−6^	8.90 × 10^−8^
		~75% necrotic cells (II)	5.50 × 10^−3^	1.40 × 10^−6^	1.30 × 10^−7^
	**Control**	(I)	6.40 × 10^−3^	2.20 × 10^−6^	1.20 × 10^−7^
		(II)	6.20 × 10^−3^	1.90 × 10^−6^	3.50 × 10^−7^
**Human Prostate**	**Cancer**	T3cNxMx, Gleason score 8(i)		2.00 × 10^−10^	4.60 × 10^−15^
		T2bNxMx, Gleason score 8(ii)		7.50 × 10^−10^	7.60 × 10^−14^
		T2cNxMx, Gleason score 6(iii)	2.50 × 10^−3^	1.70 × 10^−6^	6.20 × 10^−8^
	**Control**	(i)		2.00 × 10^−8^	4.00 × 10^−10^
		(ii)		4.30 × 10^−9^	1.80 × 10^−12^
		(iii)	4.20 × 10^−9^	1.70 × 10^−10^	8.00 × 10^−15^

**Table 2:** The total water fraction, *h*, and the remaining water fraction after BDS measurement, *h*_BDS_, for different tissues.

Type	Stress	*h*	*h*_BDS_
Rat cerebellum	DECT exposure	0.79	0.65
		0.80	0.61
	Control	0.81	0.64
		0.80	0.67
Rat skin	CONTROL fresh	0.47	0.42
	control frozen	0.49	0.43
	γ-radiation	0.55	0.49
	Control	0.57	0.50
Human lung	Adenocarcinoma	0.81	0.61
		0.79	0.66
	Control	0.77	0.60
		0.77	0.67

## Abbreviations

BDS: Broadband dielectric spectroscopy
DECT: Digital enhanced cordless telecommunication
HSP: Heat shock proteins
IR: Ionizing radiation
NIR: Non-ionizing radiation
RF-EMR: Radiofrequency electromagnetic radiation
